# Minimally invasive plate osteosynthesis with a locking compression plate is superior to open reduction and internal fixation in the management of the proximal humerus fractures

**DOI:** 10.1186/1471-2474-15-206

**Published:** 2014-06-16

**Authors:** Tao Lin, Baojun Xiao, Xiucai Ma, Dehao Fu, Shuhua Yang

**Affiliations:** 1 Department of Orthopedics, Union Hospital of Tongji Medical College, Huazhong University of Science and Technology, Wuhan 430022, Hubei Province, China

**Keywords:** Proximal humeral fractures, Minimally invasive, MIPO, Locking compression plate

## Abstract

**Background:**

The use of minimally invasive plate osteosynthesis (MIPO) via anterolateral deltoid splitting has good outcomes in the management of proximal humerus fractures. While using this approach has several advantages, including minimal soft tissue disruption, preservation of natural biology and minimal blood loss, there is an increased risk for axillary nerve damage. This study compared the advantages and clinical and radiological outcomes of MIPO or open reduction and internal fixation (ORIF) in patients with proximal humerus fractures.

**Methods:**

A matched-pair analysis was performed, and patient groups were matched according to age (±3 years), sex and fracture type. Forty-three pairs of patients (average age: MIPO, 63 and ORIF, 61) with a minimum follow-up of 12 months were enrolled in the study group. The patients were investigated radiographically and clinically using the Constant score.

**Results:**

The MIPO technique required less surgery time and caused less blood loss compared to ORIF (p < 0.01). In addition, MIPO required a smaller incision, resulted in less scarring, and was cosmetically more appealing and acceptable to female patients than ORIF. Following MIPO, patients had better functional results at 3 and 6 months, with better outcomes, less pain, higher satisfaction in activities of daily living, and a higher range of motion when compared to ORIF (p < 0.05). Fracture configuration, according to the AO/ASIF(Association for the Study of Internal Fixation) fracture classification, did not significantly influence the functional results. The complication rate was comparable between both groups.

**Conclusion:**

The use of MIPO with a locking compression plate in the management of proximal humerus fractures is a safe and superior option compared to ORIF.

## Background

There are a variety of surgical options for the treatment proximal humerus fractures, including open reduction internal fixation (ORIF), intramedullary device fixation, external fixation and hemi arthroplasty. Of these, ORIF is the most commonly used technique for the majority of fractures [1-5]. However, there is much debate on what method or technique for ORIF is optimal, and the decision is often based on the fracture configuration and surgical experience [6-12]. Recent literature has indicated that intramedullary nailing is most suitable for managing two-part proximal humerus fractures. Most surgeons agree that ORIF with a plate is the ideal technique for managing comminuted and 3 or 4 part fractures [13]. This particular technique can also be performed with minimally invasive techniques, which is known as minimally invasive plate osteosynthesis (MIPO).

Previous studies have shown that the traditional deltopectoral approach to the proximal humerus provides limited access to the posterolateral aspect of the shoulder and that the visualization and reduction of a large retracted greater tuberosity fragment may be difficult [11,14-18]. The deltopectoral approach requires extensive soft tissue dissection and muscle retraction to gain adequate exposure to the lateral aspect of the humerus [10,11,14,16,18-20]. This can cause further devascularization of fracture fragments during dissection and plating, leading to the disruption of critical blood supplies to the humeral head [10,18-20]. The deltoid splitting approach, which is an alternative method, provides good visualization of the posterolateral aspect of the shoulder without extensive soft tissue dissection or forcible retraction; however, there is an increased risk of injuring the axillar nerve as compared to the conventional deltopectoral approach [16,21,22]. Recently, many studies have demonstrated the superiority of MIPO techniques via anterolateral deltoid splitting combined with skin incisions for the management of proximal humerus fractures [15,17,19,21-28]. This method is a minimally invasive technique, leading to less soft tissue injury, decreased postoperative pain, and decreased functional loss. In addition, MIPO allows for the visualization of the axillary nerve [12,14,16,22]. Thus, MIPO is a safe and effective method for the treatment of proximal humerus fractures [14,19,20,22].

Despite these results, a consensus amongst orthopedic surgeons on the best treatment for proximal humerus fractures has not been determined [7,28]. Although many of the complications associated with MIPO are related to incorrect surgical technique, many surgeons still prefer to use the conventional ORIF with the deltopectoral approach [29]. The objective of this study was to compare MIPO using a locking compression plate and ORIF using a deltopectoral approach in the management of proximal humerus fractures. We compared surgical advantages of each technique, radiograph outcomes, the incidence of nerve injury, and functional deficits during a one year follow-up period.

## Methods

### Patient information

This was a retrospective case control study including two groups. Between September 2007 and April 2012, 184 patients with displaced proximal humerus fractures were treated with LCP (Locking Compression Plate) according to Neer criteria in Union hospital, Tongji Medical College, Huazhong University of Science and Technologe. Of these, 118were treated with ORIF via a traditional deltopectoral approach between September 2007 and June 2010, while 66 were treated with MIPO via anterolateral deltoid splitting between June 2010 and April 2012. Patients with pathological fractures, head split fractures, open fractures, fractures with primary neurovascular damage and cases lost to follow-up were excluded from the study. After these exclusions, 158 patients remained in the study. Of these 158 patients, 86 (43 pairs) were selected for a retrospective matched-paired analysis according to age (±3 years), gender, and fracture type with a minimum follow-up of 12 months (range, 12–17months). Thus, inclusion bias could be excluded.

The median age of the MIPO group was 63 years compared to 61 years in the ORIF group. The distribution of age and sex by group was 27 (62%) women and 16 (38%) men in the MIPO group, and 31 (72%) women and 12 (28%) men in the ORIF group. According to the AO/ASIF classification system, the most frequent type of fracture was type B (n = 46; 53.5%), with 19(22.1%) type A and 21 (24.4%) type C fractures. There were 10 type A, 24 type B and 9 type C fractures in the MIPO group, and there were 9 type A, 22 type B and 12 type C fractures in the ORIF group (Table 1).

**Table 1:** Patient demographics

**Characteristic**	**Treatment**		**P value**
	**MIPO (n = 43)**	**ORIF (n = 43)**	
Gender			0.357
Female	27 (62%)	31 (72%)	
Male	16 (38%)	12 (28%)	
Age, years	63 ± 14	61 ± 12	0.414
AO classification			0.753
type A	10 (23.2%)	9 (20.9%)	
type B	24 (55.8%)	22 (51.2%)	
type C	9 (20.9%)	12 (27.9%)	

### Implant

The LCP plate (titanium; thickness: 4.2 mm; width: 12 mm; length: 105-231 mm; Double Engine Medical Material Company, China) was anatomically pre-contoured with threeto ten holes on the plate shaft and nine holes for head screws. The proximal suture holes were applied to secure the tuberosity fragments and the plate.

### Surgical technique

**Figure 1:**
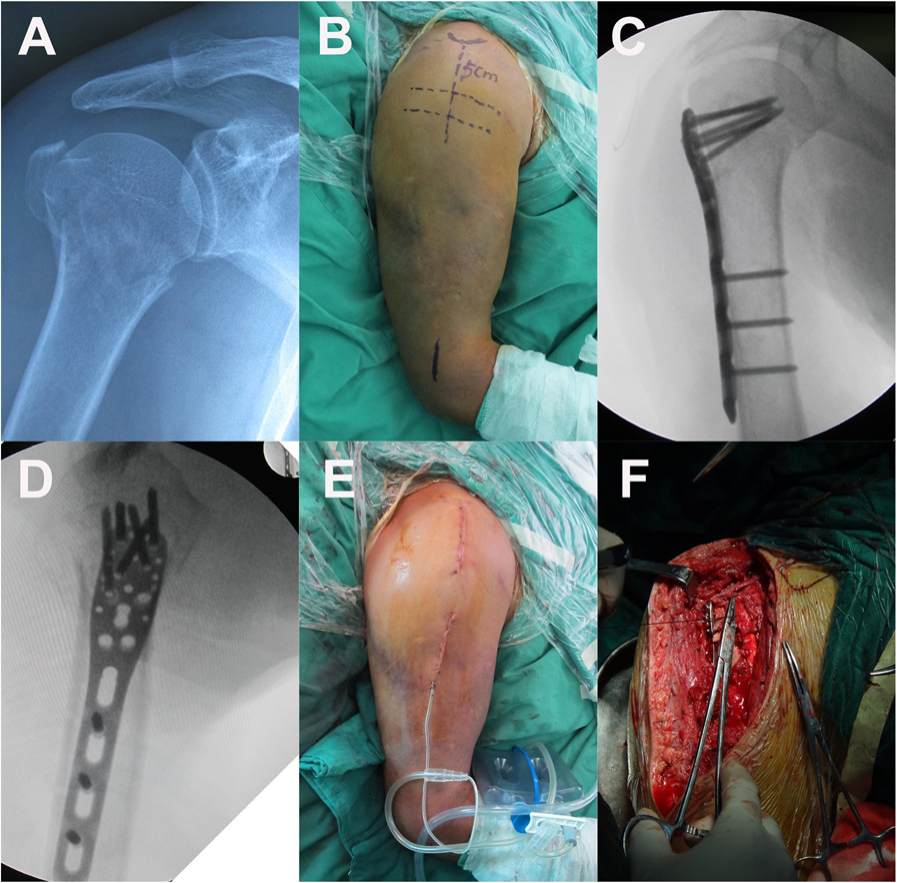
**A, Preoperative radio-graph of a70-year-old man with a displaced 3-part fracture (AO 11-B1) of the humeral head. B**, Two marker lines perpendicular to the palpable shaft of the humerus. The first line was5cm distal to the tip of the acromion and another line was2cm further distal to the first line. The area between these two lines was considered an unsafe zone. **C** and **D**, Postoperativeradiographshows proper placement of plate and screws. **E**, Theminimally invasive approach shows that two small incisions with a skin bridge over the palpated axillary nerve. **F**, Thedeltopectoral approachrequired an approximately 14cmin the ORIF group.

In both the MIPO and ORIF groups, the patients were positioned in the beach-chair or supine position to allow two plane intraoperative C-arm image intensifier views [8,11,12,16,18,19,30-32]. All of the procedures were performed under general anesthesia with administration of a broad-spectrum antibiotic prophylaxis [16,18,22,30]. In the ORIF group, ORIF was performed using a standard deltopectoral approach with a LCP. The 12-14 cm incision started at the tip of the coracoid process and ran laterally in the direction of the insertion of the deltoid muscle (Figure 1F). Reduction was enabled with a K-wire under fluoroscopy according to the landmarks of the long head of biceps, the greater and lesser tubercles, and the intertubercular groove [26,30]. After the fracture was anatomically reduced, a LCP was placed 1 cm posterior to the intertubercular groove [11,30] and 1 cm distal to the tip of the greater tubercle [15,27].

In the MIPO group, the anterolateral deltoid splitting approach was utilized, and the tip of the acromion was palpated and used as a landmark [14,19]. A line perpendicular to the palpable shaft of the humerus was drawn 5 cm distal to the tip of the acromion. A second parallel line was drawn 2 cm further distal to the first line (Figure 1B). The area between these two lines contained the axillary nerve and was considered the unsafe zone. A longitudinal incision was made on the lateral side of the humerus starting from the lateral acromial border and ending distally 5 cm was to access the proximal humerus, the greater tuberosity and the humeral head (Figure 1E). To insert distal screws, a distal incision was made 7 cm distal to the acromion and was approximately 2.5-3.5 cm long (Figure 1E). In most cases, good reduction was achieved by applying axial traction on the humerus and pulling the rotator cuff [6,8,22,23,33]. In some cases, indirect reduction techniques, such as ligamentotaxis, were used. Plate reduction was performed in cases of a valgus displaced fracture configuration [22,28]. The LCP was placed proximally below the apex of the greater tuberosity to maintain reduction. If non-absorbable sutures were used, they were secured to the suture wires holes in the LCP [6,11,16,19,21-23,26,28,33]. The plate was anchored proximally with multiple angled stable screws into the humeral head fragment. After removing the aiming arm, the non-absorbable sutures were tightened to the LCP [21-23].

Patients in both groups had individual patient-related postoperative management. In the majority of cases, the patients’ arm was placed in a sling for a maximum of two weeks. Passive and active range of motion exercises were started after surgery, depending on pain and activity level [6,11].

### Ethics and consent

This study has been performed in compliance with the Helsinki Declaration and has been granted an exemption from Hospital’s Ethics Committee of Union Hospital of Tongji Medical College. The patients were informed and have written informed consent; all patients were over 18 years old. The classic cases in this article have been undertaken with the patient's consent.

### Data analysis

We used SPSS 18 statistical software for Windows for all analyses. For normally distributed data (patient age, time between fracture and fixation, operative time, blood loss, hospital stay, and follow-up time), an independent sample t-test was used. For data that was not normally distributed data (AO classification and the Constant shoulder score), the Mann–Whitney rank sum test was used. For categorical data (gender and fracture pattern), a chi-square test was used. A P-value < 0.05 was considered statistically significant.

## Results

### Follow up

We performed clinical and radiographic assessments 3, 6 and 12 months after surgery [6,11]. At each follow-up, the Constant score was used to assess shoulder function. Standardized X-rays were obtained in anteroposterior and transscapular views to evaluate fracture healing, avascular necrosis, placement of the plate, and quality of reduction. Complications were evaluated based on follow-up radiographs and a retrospective chart review of the patients’ medical records to determine the incidence of humeral head necrosis, delayed union, implant failure or a neurological deficit.

To perform the retrospective matched-paired analysis, we selected patients according to age (±3 years), gender and fracture types. The patient demographics of 86 patients are listed in Table 1. The analysis revealed no significant differences in group demographics, including AO/ASIF classification and mean age (p > 0.05). However, there was a higher female ratio in the ORIF group compared to the MIPO group (p > 0.05). The average duration between trauma and surgery in the MIPO group was 5 days and 6.3 days in the ORIF group. In addition, MIPO required less surgery time and resulted in less blood loss (both p < 0.01). The union rate at 6 months was 93% in the MIPO group and 97.7% in the ORIF group (Table 2).

**Table 2:** Surgical and follow-up data

**Characteristic**	**Treatment**		**P value**
	**MIPO (n = 43)**	**ORIF (n = 43)**	
Average length of surgery in min	71 ± 8.7	79 ± 11.7	0.0007
Average length of hospital stay in days	**6.8** ± 1.8	**7.7** ± 1.5	0.046
Average blood loss (ml)	126 ± 54.8	213 ± 68.4	0
Average duration between trauma and surgery(day)	**5.0** ± 1.9	**6.3** ± 1.8	0.0012
Follow-up(months)	12.6 ± 1.4	13.1 ± 0.9	0.067
The rate of union (at 6 months)	**93%**	97.7%	

**Figure 2:**
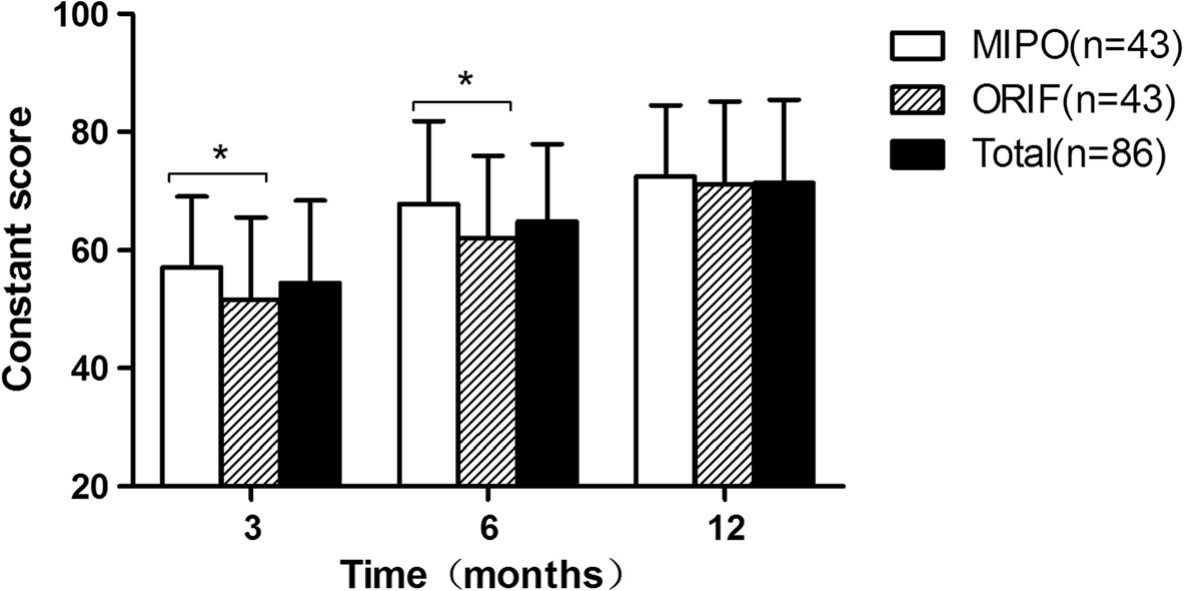
**Mean value of Constant score at each follow up.** Mean values of the Constant score for all patients (total), MIPO and ORIF at 3, 6, and 12 months (*p < 0.05).

**Figure 3:**
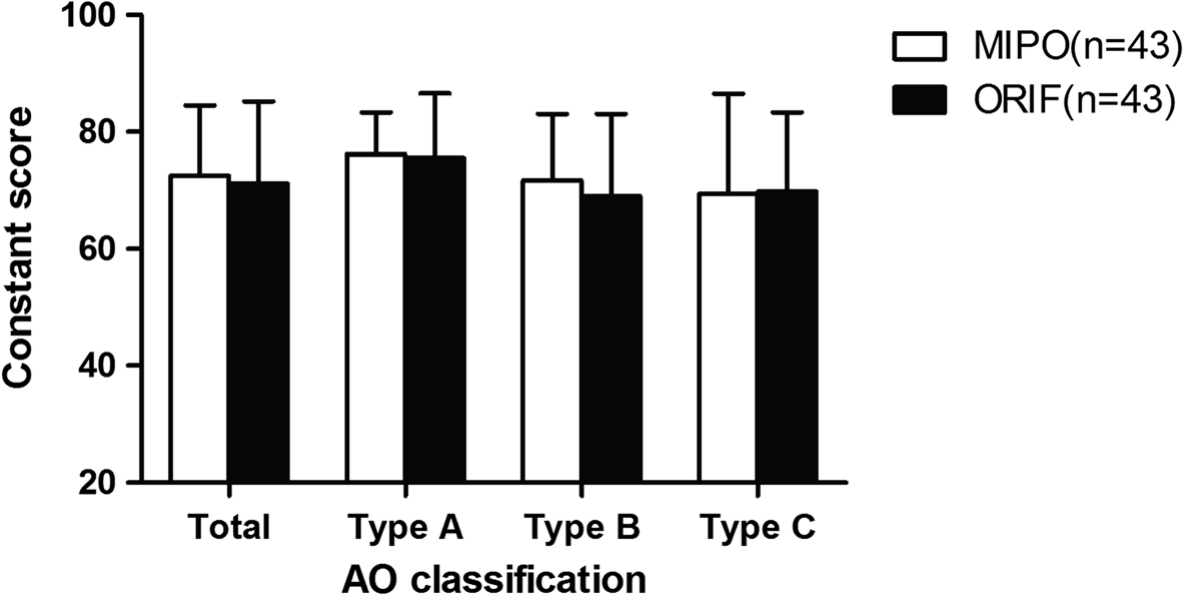
**Average scores for the fracture types based on the AO/ASIF fracture classification after12months of follow-up.** Type Afractures had the highest average Constant score in both groups, followed by type B and C fractures. There were no significant differences toward the type of fracture and each type between groups (p = 0.205).

The Constant score was higher in the MIPO group at the 3 and 6 month follow-up time points compared to the ORIF group (P =0.033 and P = 0.043) (Figure 2). At the 12 month follow-up, the Constant score was not statistically significant (P = 0.065) (Figure 2). In addition, patients in the MIPO group experienced significantly less pain, higher satisfaction in activities of daily living, and greater range of motion at the 3 and 6 months follow-up time points (p < 0.05). The level of strength was not significantly different at these time points (p > 0.05). At the 12 month follow-up, there were no significant differences in both groups (Table 3).At the 12 month follow-up, type A fractures had the highest average Constant score in both groups (MIPO: 76.2 ± 7.1, ORIF: 75.6 ± 10.8), followed by type B fractures (MIPO: 71.7 ± 11.3, ORIF 69.0 ± 14.2; ORIF: 69. ±13.9) and type C fractures (MIPO: 69.4 ± 17.1, ORIF: 69.8 ± 13.2) (Figure 3). The type of fracture between groups was not significantly different (p = 0.205).

**Table 3:** Subjective parameters of the constant score

**Score**	**3 months**			**6 months**			**12 months**		
	**MIPO (n = 43)**	**ORIF (n = 43)**	**P value**	**MIPO (n = 43)**	**ORIF (n = 43)**	**P value**	**MIPO (n = 43)**	**ORIF (n = 43)**	**P value**
Pain	8.8 ± 3.4	7.2 ± 2.9	0.020	12.8 ± 3.1	11.3 ± 3.3	0.007	13.3 ± 2.8	12.8 ± 3.2	0.373
ADL	14.5 ± 3.0	12.8 ± 2.8	0.012	15.6 ± 3.3	14.0 ± 2.7	0.039	17.1 ± 3.1	16.0 ± 2.9	0.073
ROM	25.6 ± 3.9	23.6 ± 4.0	0.022	29.7 ± 4.1	26.9 ± 4.1	0.002	30.3 ± 3.6	29.7 ± 4.1	0.438
Strength	8.23 ± 2.3	7.9 ± 2.2	0.536	9.7 ± 3.1	10.2 ± 2.9	0.195	11.5 ± 3.2	12.3 ± 3.4	0.287
Total	57.1 ± 12	51.6 ± 14	0.033	67.7 ± 14	62.0 ± 14	0.040	72.5 ± 12	71.2 ± 14	0.652

*ADL* activities of daily living; *ROM* range of motion.

In the MIPO group, 5/43 patients experienced complications and 4/43 patients experienced complications in the ORIF group (Table 4). No patients developed wound infection and nonunion after one year of follow-up in both groups. In 3 patients (group MIPO, 2 patients, type B and C; group ORIF, 1 patient, type B), the fracture collapsed after 3 months, leading to a varus malalignment. These patients developed loss of reduction and underwent reoperation either by reosteosynthesis combined with cancellous grafting or by joint replacement. One patient in the MIPO group presented with clinical signs of axillary injury, which was characterized by poorly localized posterior shoulder pain, parenthesis over the lateral aspect of the shoulder, and deltoid muscle weakness. Axillary nerve injury was confirmed on electromyography examination. However, there was no functional impairment when the patient was assessed at one year follow-up. One plate in the MIPO group and two plates in the ORIF group were removed due to subacromial impingement after radiographs confirmed fracture union at about 5 months. In the ORIF group, one patient underwent reoperation to change a perforated screw 3 months after the initial operation.

**Table 4:** Complications after one year of follow-up

**Complications**	**MIPO (n = 43)**	**ORIF (n = 43)**
Complications rate	5 (11.6%)	4 (9.3%)
Reoperations (%)	2 (4.6%)	2 (4.6%)
Varus displacement	1	
Second loss of reduction	2	1
Nerve lesions	1	
Impingement rates	1	2
Screw perforation		1

## Discussion

The objective of this study was to compare MIPO and ORIF with a LCP plate to treat proximal humerus fracture. Our retrospective comparative study showed that the MIPO technique was superior to ORIF in the management of proximal humerus fractures. First, the MIPO group required less surgery time, resulted in less blood loss, and the patients required a relatively shorter hospital stay. Second, the MIPO group had better shoulder function at 3 and 6 months, with less pain, higher satisfaction in activities of daily living and greater range of motion (p < 0.05). The complication rate was comparable between both groups (MIPO:11.6%;ORIF:9.3%).

The MIPO technique provides good visualization of the posterolateral aspect of the shoulder via a small incision without extensive soft-tissue dissection or forcible retraction. Thus, it is relatively easy to perform a reduction of a large greater tuberosity fragment under direct vision and significantly reduces the intra-operative time. These findings were in accordance to previous reports [11,19,23,28].

In this study, Constant scores based on fracture type were similarly distributed in both groups. In general, type A fractures had the highest average Constant score, followed by type B and C fractures. However, when subgroup analysis was carried out, the Constant scores were higher for type A and B fractures treated with MIPO. This was in contrast with previous reports by Hepp et al. [11].

In this study, the technique (MIPO/ORIF) employed had no significant influence on the final functional muscle weakness and shoulder range of motion. In addition, these findings were independent of an axillary nerve injury. These findings are not consistent with the findings from Hepp et al. [11]. Their study suggested that the ORIF technique had a higher deltoid muscle level at 3 and 6 months follow up. In addition, there was less soft tissue disruption in the MIPO group, most likely leading to a greater range of movement when compared to the ORIF group. Taken together, these findings indicate that the MIPO technique is safe and has a low risk of axillary nerve injury for treating proximal humerus fractures.

Previous anatomical studies have revealed that axillary nerve lesions occur between 5.58 cm to 6.66 cm distal to the lateral acromion [14,34,35]. In this study, to prevent the axillary nerve damage, we split the deltoid no more than 5 cm distal to the mid-acromion in any given vertically neutral position [16,35]. For the second incision to place distal screws, we recommend a starting deltoid split point at least 7 cm distal to the acromion, while Cheung [35] suggests 9 cm. In our study, none of the patients required an extension of the incision. Axillary nerve palsy in one patient was most likely due to sliding of the LCP plate. A study by Visser et al. [36,37] showed that axillary nerve lesions were more frequent in proximal humeral fractures, with an overall incidence of 58% and 62%. It should be noted that clinical exam is not reliable in detecting nerve injuries, and an electromyography investigation is recommended for an accurate diagnosis [36,37].

Moreover, this study revealed that the surgical approach did not influence the complication rate and/or radiological outcomes. The complication rate of the MIPO group was less than 12%, which is comparable to previous reports [21,23,30,31,38]. In the literature, the main complications with LCP are implant-related, including impingement, intra-articular screw perforation and the proximal screw loosening [21,22,31,33,38].

In our study, the major complication was secondary loss of reduction following a varus collapse of the fracture. This also resulted in subacromial impingement due to a reduced acromio-humeral distance. In these three patients, there was loss of medial hinge integrity due to impaction and osteoporosis, causing the fractures to be unstable. Recent studies have demonstrated a direct association between medial support and subsequent reduction loss [30,38]. In the MIPO group in this study, wound healing occurred faster and there was minimal scaring following surgery. Thus, patients might have engaged in early full weight bearing activities and functional exercises, leading to delayed healing.

The second major complication was subacromial impingement, which occurred in one plate in the MIPO group and two plates in the ORIF group. The plates were removed after radiograph confirmation of the fracture union at about 5 months. At the last follow-up, the patients achieved optimal functional outcomes with good range of motion.

In our study, the most frequent type of fracture was type B in both groups. The mean Constant score of type B fractures was more than in type A and type C fractures. These findings are similar to the report by Röderer and Brorson et al. [32,39], although our overall complication rate was much lower.

There are some limitations in our study. First, our study was retrospective in design. However, our study provided long-term results and outcomes of patients undergoing MIPO compared to ORIF. Second, the study was conducted at a single center with different surgeons. The surgeons were experienced and had expertise in MIPO and ORIF. Third, patients treated with MIPO underwent surgery 1–3 years later than the patients who were treated with ORIF. All the other parameters were similar between the two groups.

## Conclusion

This study shows that MIPO with LCP requires less surgery time, causes less blood loss, shortens hospital stay,results in less scarring, and is cosmetically more appealing and acceptable to female patients compared to ORIF with LCP. Further, MIPO with LCP provides good functional results and has less morbidity at one year follow-up. MIPO with LCP for proximal humerus fractures is a safe and favorable option compared to ORIF with LCP.

## Abbreviations

LCP: Locking compression plate; MIPO: Minimally invasive plate osteosynthesis; ORIF: Open reduction and internal fixation; ADL: Activities of daily living; ROM: Range of motion.

## Competing interests

The authors have declared that no competing interest exists.

## Authors’ contributions

All authors have made substantial contributions to design, acquisition of data, and analysis and interpretation of data. DF and SY conceived the study. TL, XM and DF were responsible for the design and the statistical analysis. TL and BX were involved in drafting the manuscript or revising it critically for important intellectual content. All the authors read and approved the final manuscript. DF and SY have given final approval of the version to be published.

## Pre-publication history

The pre-publication history for this paper can be accessed here:

http://www.biomedcentral.com/1471-2474/15/206/prepub
